# Associations between digital maturity in health and primary health care performance, 109 countries

**DOI:** 10.2471/BLT.24.292706

**Published:** 2025-09-25

**Authors:** Lena Kan, Yoko Shimada, Abdulaziz Mohammed Hussen, Arisa Shichijo Kiyomoto, Shivani Pandya, Patricia Mechael, Binyam Tilahun, Meredith Kimball, Marelize Gorgens, Malarvizhi Veerappan, Ethan Wong, Smisha Agarwal

**Affiliations:** a Center for Global Digital Health Innovation, Johns Hopkins Bloomberg School of Public Health, Johns Hopkins University, 615 North Wolfe St, Baltimore, Maryland, MD 21205, United States of America (USA).; b Center for Digital Health and Implementation Sciences, University of Gondar, Gondar, Ethiopia.; c World Bank, Washington, DC, USA.; d Gates Foundation, Seattle, USA.

## Abstract

**Objective:**

To investigate associations between digital maturity in health and primary health care performance globally.

**Methods:**

We conducted a search of publicly available data on digital maturity in health and primary health care performance for the 194 World Health Organization Member States. We identified 14 indicators of digital maturity in health, covering seven core subcomponents. A digital maturity in health index was derived from these indicators. Primary health care performance was assessed using the universal health coverage effective coverage index.

**Findings:**

Digital maturity in health data were missing for 85 of the 194 countries, with considerable variation across subcomponents. The remaining 109 countries were divided into four types by digital maturity in health index. We identified countries leading or lagging in digital maturity and highlighted the strongest and weakest subcomponents. Overall, there was a strong, nonlinear, positive correlation between digital maturity in health and primary health care performance (Spearman correlation: 0.85). However, there were notable exceptions, which indicates digital maturity can enhance primary health care but is not necessary for its improvement. The relationship between health-care expenditure and digital maturity in health and primary health care performance varied among countries with similar spending and digital maturity.

**Conclusion:**

Overall, primary health care performance was positively associated with digital maturity in health and health-care expenditure. However, some countries had a strong primary health care system despite low digital maturity, and some had high digital maturity but a weak primary health care system. The study’s findings could help policy-makers prioritize investment in digital health.

## Résumé

**Objectif:**

Étudier les associations entre la maturité numérique dans le domaine de la santé et les performances des soins de santé primaires à l’échelle mondiale.

**Méthodes:**

Nous avons analysé des données accessibles au public concernant la maturité numérique dans le domaine de la santé et les performances des soins de santé primaires pour les 194 États membres de l’Organisation mondiale de la Santé. Nous avons identifié 14 indicateurs de maturité numérique dans le domaine de la santé, couvrant sept sous-composantes essentielles. Un indice de maturité numérique dans le domaine de la santé a été dérivé de ces indicateurs. Les performances des soins de santé primaires ont été évaluées à l’aide de l’indice de couverture efficace des soins de santé universels.

**Résultats:**

Les données relatives à la maturité numérique dans le domaine de la santé faisaient défaut pour 85 des 194 pays, avec des variations considérables entre les sous-composantes. Les 109 pays restants ont été divisés en quatre types selon leur indice de maturité numérique dans le domaine de la santé. Nous avons identifié les pays en avance ou en retard en matière de maturité numérique et avons mis en évidence les sous-composantes les plus fortes et les plus faibles. Dans l’ensemble, il existait une forte corrélation positive et non linéaire entre la maturité numérique dans le domaine de la santé et les performances des soins de santé primaires (corrélation de Spearman: 0,85). Des exceptions notables subsistaient, ce qui indique que la maturité numérique est de nature à améliorer les soins de santé primaires, sans être indispensable à leur amélioration. La relation entre les dépenses de santé et la maturité numérique dans le domaine de la santé et les performances des soins de santé primaires variait parmi les pays aux dépenses et à la maturité numérique similaires.

**Conclusion:**

Dans l’ensemble, les performances des soins de santé primaires présentaient une association positive à la maturité numérique dans le domaine de la santé et aux dépenses de santé. Toutefois, certains pays disposaient d’un système robuste de soins de santé primaires malgré une faible maturité numérique, tandis que d’autres présentaient une maturité numérique élevée, mais un faible système de soins de santé primaires. Les conclusions de cette étude pourraient aider les décideurs politiques à hiérarchiser les investissements dans la santé numérique.

## Resumen

**Objetivo:**

Investigar las asociaciones entre la madurez digital en salud y el desempeño de la atención primaria de salud a nivel mundial.

**Métodos:**

Se realizó una búsqueda de datos disponibles públicamente sobre madurez digital en salud y desempeño de la atención primaria de salud para los 194 Estados Miembros de la Organización Mundial de la Salud. Se identificaron 14 indicadores de madurez digital en salud, que abarcan siete subcomponentes fundamentales. A partir de estos indicadores, se elaboró un índice de madurez digital en salud. El desempeño de la atención primaria de salud se evaluó mediante el índice de cobertura efectiva de la cobertura sanitaria universal.

**Resultados:**

Faltaban datos de madurez digital en salud para 85 de los 194 países, con una variación considerable entre subcomponentes. Los 109 países restantes se clasificaron en cuatro tipos según el índice de madurez digital en salud. Se identificaron países con mayor o menor desarrollo en madurez digital y se destacaron los subcomponentes más sólidos y más débiles. En general, se observó una correlación positiva, no lineal y sólida entre la madurez digital en salud y el desempeño de la atención primaria de salud (correlación de Spearman: 0,85). Sin embargo, se registraron excepciones notables, lo que indica que la madurez digital puede potenciar la atención primaria de salud, aunque no es indispensable para su mejora. La relación entre el gasto sanitario, la madurez digital en salud y el desempeño de la atención primaria de salud varió entre países con niveles similares de gasto y madurez digital.

**Conclusión:**

En conjunto, el desempeño de la atención primaria de salud se asoció positivamente con la madurez digital en salud y el gasto sanitario. No obstante, algunos países presentaban un sistema sólido de atención primaria de salud pese a su baja madurez digital, mientras que otros mostraban alta madurez digital pero un sistema de atención primaria débil. Los hallazgos del estudio podrían ayudar a los responsables de la elaboración de políticas a priorizar la inversión en salud digital.

## ملخص

الغرض فحص الارتباطات بين النضج الرقمي في مجال الصحة وأداء الرعاية الصحية الأولية على مستوى العالم.

الطريقة قمنا بإجراء بحث في البيانات المتاحة للعامة حول النضج الرقمي في مجال الصحة وأداء الرعاية الصحية الأولية للدول الأعضاء في منظمة الصحة العالمية، والبالغ عددها 194 دولة. وقمنا بتحديد 14 مؤشرًا للنضج الرقمي في مجال الصحة، تغطي سبعة مكونات فرعية أساسية. وتم استنتاج مؤشر للنضج الرقمي في مجال الصحة من خلال هذه المؤشرات. كما تم تقييم أداء الرعاية الصحية الأولية باستخدام مؤشر الفعالية للتغطية الصحية الشاملة.

النتائج لم تتوفر بيانات النضج الرقمي في مجال الصحة في 85 دولة من أصل 194 دولة، مع وجود تنوع كبير في المكونات الفرعية. وتم تقسيم الدول الـ 109 المتبقية إلى أربعة أنواع حسب مؤشر النضج الرقمي في مجال الصحة. وحددنا الدول الرائدة أو المتأخرة في النضج الرقمي، وسلطنا الضوء على أقوى وأضعف المكونات الفرعية. وبشكل عام، كان هناك ارتباط إيجابي وقوي وغير خطي بين النضج الرقمي في مجال الصحة وأداء الرعاية الصحية الأولية (معامل ارتباط سبيرمان: 0.85). ومع ذلك، كانت هناك استثناءات ملحوظة، مما يشير إلى أن النضج الرقمي يُمكنه أن يُعزز الرعاية الصحية الأولية، ولكنه ليس ضروريًا لتحسينها. وتفاوتت العلاقة بين إنفاق الرعاية الصحية والنضج الرقمي في الصحة وأداء الرعاية الصحية الأولية، بين الدول ذات الإنفاق والنضج الرقمي المتشابهين.

الاستنتاج بشكل عام، ارتبط أداء الرعاية الصحية الأولية ارتباطًا إيجابيًا بالنضج الرقمي في الصحة والإنفاق عليها. إلا أن بعض الدول كان لديها أنظمة رعاية صحية أولية قوية بالرغم من ضعف النضج الرقمي لديها، بينما كان لدى بعض دول أخرى نضج رقمي مرتفع، ولكن نظام الرعاية الصحية الأولية ضعيف. ويمكن أن تساعد نتائج الدراسة واضعي السياسات على تحديد أولويات الاستثمار في الصحة الرقمية.

## 摘要

**目的:**

旨在研究全球医疗卫生数字化成熟度与初级卫生保健绩效之间的关系。

**方法:**

我们检索了与 194 个世卫组织成员国的医疗卫生数字化成熟度和初级卫生保健绩效相关的公开可用数据。我们确定了涵盖七个核心子组成部分的 14 个医疗卫生数字化成熟度指标。我们根据这些指标推导得出了医疗卫生数字化成熟度指数。通过使用全民健康覆盖相关有效覆盖指数，我们评估了初级卫生保健绩效。

**结果:**

在这 194 个国家中，有 85 个国家缺失医疗卫生数字化成熟度数据，且在各子组成部分之间差异显著。其余 109 个国家可根据医疗卫生数字化成熟度指数分为四类。我们确定了在数字化成熟度方面处于领先或落后地位的国家，并强调了其最强和最弱的子组成部分。总体而言，医疗卫生数字化成熟度与初级卫生保健绩效之间存在很强的非线性正相关关系（斯皮尔曼相关系数：0.85）。但是，也存在明显例外的情况，这说明数字化成熟度有助于优化初级卫生保健系统，但其也并非改善初级卫生保健的必要条件。有些国家的卫生保健开支与数字化成熟度虽然类似，其卫生保健开支与医疗卫生数字化成熟度和初级卫生保健绩效之间的关系却截然不同。

**结论:**

总体而言，初级卫生保健绩效与医疗卫生数字化成熟度和卫生保健开支之间呈正相关关系。但是，虽然有些国家的数字化成熟度较低，但其却拥有非常完善的初级卫生保健系统，而有些国家的数字化成熟度较高，其初级卫生保健系统却比较薄弱。该研究的结果可为政策制定者优先规划数字卫生保健领域的投资提供帮助。

## Резюме

**Цель:**

Изучить связь между цифровой зрелостью в вопросах здоровья и эффективностью первичной медико-санитарной помощи в мировом масштабе.

**Методы:**

Был проведен поиск по общедоступным данным, касающимся цифровой зрелости в вопросах здоровья и эффективности первичной медико-санитарной помощи для 194 государств-членов Всемирной организации здравоохранения. Авторы установили 14 индикаторов цифровой зрелости в вопросах здоровья, которые охватывают семь ключевых субкомпонентов. На основании этих индикаторов вычислялся показатель цифровой зрелости в вопросах здоровья. Показатели эффективности первичной медико-санитарной помощи оценивались с использованием показателя всеобщего эффективного охвата услугами здравоохранения.

**Результаты:**

Данные относительно цифровой зрелости в вопросах здоровья отсутствовали для 85 из 194 стран, и наблюдались существенные вариации по субкомпонентам. Остальные 109 стран были разделены на четыре типа по показателю цифровой зрелости в вопросах здоровья. Авторы определили, какие страны являются ведущими или отстающими в вопросах цифровой зрелости, а также выявили наиболее сильные и слабые субкомпоненты. В целом наблюдалась сильная нелинейная позитивная корреляция между цифровой зрелостью в вопросах здоровья и эффективностью первичной медико-санитарной помощи (корреляция Спирмана: 0,85). Однако были и заметные исключения, которые указывают на то, что цифровая зрелость может повысить качество первичной медико-санитарной помощи, но не является необходимой для ее улучшения. Связь межу затратами на здравоохранение и цифровой зрелостью в вопросах здоровья, а также эффективностью первичной медико-санитарной помощи была различной для стран со сходным уровнем расходов и показателем цифровой зрелости.

**Вывод:**

В целом эффективность первичной медико-санитарной помощи была прямо связана с цифровой зрелостью в вопросах здоровья и затратами на здравоохранение. Однако в некоторых странах имелась сильная система первичной медико-санитарной помощи, несмотря на низкий уровень цифровой зрелости, а некоторые имели высокий уровень цифровой зрелости при слабой системе первичной медико-санитарной помощи. Результаты исследования могут помочь лицам, принимающим решения политического характера, уделять приоритетное внимание инвестициям в цифровое здравоохранение.

## Introduction

A well-functioning primary health care system that provides high-quality and affordable services is essential for achieving universal health coverage (UHC). Although primary health care delivers nearly 90% of essential health services,^1^^–^^3^ it remains underfunded: in 2025, the annual investment shortfall was estimated to be between 200 and 370 billion United States dollars (US$).^4^ This funding gap exacerbates health inequalities, particularly in low- and middle-income countries.^5^^,^^6^ In 2019, the World Health Organization (WHO) reported a 18.1-year gap in life expectancy between the poorest and richest countries.^7^ These substantial variations in primary health care performance highlight the need for strategic investment.^6^

Over the past two decades, increased access to mobile devices and the internet has provided new opportunities for strengthening primary health care delivery and quality. By 2021, mobile broadband had reached 95% of the global population, with much of the growth occurring in low- and middle-income countries, where half the population now uses the mobile internet.^8^ The World Bank’s *Digital-in-health: unlocking the value for everyone* report emphasized the role of digital technologies in strengthening health systems and improving the effectiveness, equity and reach of health service delivery and financing.^9^ By 2024, over 120 countries had developed national digital health strategies,^8^^,^^10^ which reflects a global commitment to integrating digital technology into aspects of health services such as electronic health records, clinical decision support and diagnostics management.^11^^–^^13^

The coronavirus disease 2019 (COVID-19) pandemic accelerated interest in, and the adoption of, digital health services,^14^^,^^15^ particularly for telemedicine, supply-chain systems and health communications programmes. However, many initiatives were fragmented, lacked coordination and had limited sustainability beyond the pandemic’s acute phase. Efforts focused on vertical interventions and often lacked strategies for interoperability, sustainability or scaling up.^16^ The pandemic underscored the need for governments to ensure that investment in digital health is directed towards building a sustainable digital infrastructure capable of scaling up digitally assisted primary health care delivery and pandemic preparedness.

Although evidence is still emerging, several studies of the impact of digital technology on primary health care in low- and middle-income countries found positive associations between digital interventions and improved primary health care outcomes.^17^^–^^20^ In addition, WHO’s recommendations on digital interventions for health systems strengthening emphasize the role of implementation and contextual factors in the effective deployment and scaling up of digital interventions.^21^ Consequently, an understanding of these digital ecosystem factors is essential for ensuring that digitization leads to measurable improvements in health systems and health outcomes, and for identifying drivers of sustainable digital transformation.^16^^,^^21^^–^^24^

The aims of our study, which was part of the Exemplars in Global Health programme,^25^ were to identify drivers of digital ecosystem maturity and to examine their impact on primary health care performance in individual countries. By examining national experience globally, we sought to identify regional leaders and countries that have successfully used digital approaches for improving primary health care systems and to document transferable insights and strategies. 

## Methods

Currently, there is no standardized measure of the maturity of a country’s digital health ecosystem.^26^ Although many frameworks and indicators have been proposed, none offers a comprehensive, integrated and measurable approach.^2^^,^^27^^–^^30^ Consequently, we developed an index for digital maturity in health (hereafter referred to as a digital maturity) by selecting indicators aligned with the WHO and International Telecommunication Union’s National eHealth Strategy Toolkit,^31^ which identifies seven key building blocks for the effective deployment and scaling up of digital health interventions: (i) leadership and governance; (ii) strategy and investment; (iii) services and applications; (iv) standards and interoperability; (v) infrastructure; (vi) legislation, policy and compliance; and (vii) workforce. Details of the building blocks are available from the online repository.^32^

The digital maturity indicator selection process is outlined in the online repository.^32^ In January 2023, we conducted a review of 101 potential indicators from publicly available data sources, including the World Economic Forum’s Network Readiness Index,^33^ the GSMA Mobile Connectivity Index,^34^ and the World Bank’s GovTech Maturity Index.^35^ We identified seven subcomponents of the digital maturity index. Five were selected from the National eHealth Strategy Toolkit: (i) infrastructure; (ii) workforce; (iii) leadership and governance; (iv) strategy and investment; and (v) legislation, policy and compliance.^31^ We identified the remaining two through consultation with a technical advisory group convened for the study: (vi) gender diversity; and (vii) consumer readiness. We excluded the standards and interoperability and services and applications building blocks from the toolkit^31^ because there were no measurable indicators. The final 14 digital maturity indicators (Table 1) were selected for their relevance to the seven subcomponents and according to criteria such as the availability, recency, measurability and comparability of data. Although several of the selected indicators relate to the maturity of a country’s digital ecosystem overall, they are also relevant to the health sector.

**Table 1:** Subcomponents, indicators and data sources for a digital maturity in health index, associations between digital maturity in health and primary health care performance, 109 countries, 2023

Digital maturity in health index subcomponents and indicators^a^	Data source^c^
**Infrastructure**	
Proportion of population covered by at least a 3G mobile network	International Telecommunication Union ICT Indicators database^36^
Government online services index	United Nations E-Government Knowledgebase^37^
Proportion of population with access to electricity	World Bank^38^
**Workforce**	
Information and communication technology skill training in the education system	World Economic Forum’s Executive Opinion Survey^39^
**Leadership and governance**	
GovTech Maturity Index	World Bank^35^
**Strategy and investment**	
Investment in emerging technologies	World Economic Forum’s Executive Opinion Survey^39^
Computer software expenditure as a percentage of gross domestic product	S&P Global market intelligence^40^
**Legislation, policy and compliance**	
Information and communication technology regulatory environment	International Telecommunication Union ICT Regulatory Tracker^41^
Regulation of emerging technologies	World Economic Forum’s Executive Opinion Survey^39^
Privacy protection in law	Digital Society Project data set^42^
Global cybersecurity index	International Telecommunication Union^43^
**Gender diversity^b^**	
Gender gap ratio in social media use	We Are Social and Facebook audience insights^44^^,^^45^
Gender gap ratio for mobile phone ownership	GSMA Intelligence and Gallup World Poll^46^^,^^47^
**Consumer readiness^b^**	
Proportion of population that owns a mobile phone	GSMA Intelligence^46^

^a^ Full definitions and descriptions of the indicators for digital maturity in health are available in the data repository.^32^

^b^ The gender diversity and consumer readiness subcomponents were recommended by the study’s technical advisory group members.

^c^ All data sources were derived from index methodology reports of the World Economic Forum’s Network Readiness Index,^33^ the GSMA Mobile Connectivity Index,^34^ and the World Bank’s GovTech Maturity Index.^35^

### Technical advisory group

To guide the development of a digital maturity index, we convened a technical advisory group of 21 experts in primary health care, digital health and health systems strengthening. Group members represented multilateral and bilateral agencies, nongovernmental organizations, governments, technical support agencies, academic institutions, financial donors and local advocacy organizations. The group provided critical guidance throughout the digital maturity index development process and helped to iteratively refine subcomponents and indicators. In particular, the group identified gender diversity and consumer readiness as essential subcomponents for assessing equitable access to digital services (Table 1). The inclusion of gender diversity reflects the pivotal role of women as primary caregivers and decision-makers in family and community health. In turn, consumer readiness can act as a proxy for equitable access to, and the use of, digital services because it reflects the presence of barriers such as: (i) limited digital literacy; (ii) low trust in, and a reluctance to adopt, digital technologies; (iii) problems with affordability; and (iv) infrastructure gaps.

### Primary health care performance

To measure primary health care performance, we used the UHC effective coverage index (hereafter referred to as the performance index) developed by the Institute for Health Metrics and Evaluation.^48^ This index comprises 23 indicators across five service domains: (i) promotion; (ii) prevention; (iii) treatment; (iv) rehabilitation; and (v) palliation. In addition, the index covers five age groups: (i) reproductive and newborn; (ii) children younger than 5 years; (iii) children and adolescents aged 5 to 19 years; (iv) adults aged 20 to 64 years; and (v) adults aged 65 years or older. The index incorporates measures of the coverage of interventions and measures of health outcomes normalized on scales of 0 to 100. The index was validated by the 2019 Global Burden of Diseases, Injuries, and Risk Factors Study and found to be more comprehensive than other UHC service coverage indices.^48^

### Analysis

#### Digital maturity index

The value of each indicator was normalized on a scale of 0 to 100 to ensure standardization in deriving the digital maturity index, which was then calculated as the average of all 14 indicators. The subcomponent values were the average of their constituent indicator values, as detailed in the online repository.^32^ We created a database for the WHO Member States for which data on digital maturity were available.^49^

#### Assessing digital maturity

We identified countries for which data were missing on at least one of the 14 indicators, and analysed variations in missing data across the seven subcomponents and six WHO regions. We divided the countries with complete data available into four country types based on quartiles of their digital maturity index values: (i) emerging (index: 27.9 to 60.3); (ii) transitioner (index: 60.4 to 69.8); (iii) advanced (index: 69.9 to 78.3); and (iv) leader (index: 78.4 to 92.7). Using this categorization, we highlighted countries that were leading or lagging in digital maturity or primary health care performance, and examined differences across subcomponents and country types.

#### Digital maturity, UHC and health spending

We plotted each country's performance index against its digital maturity index to investigate the relationship between these two variables (details available in the online repository),^32^ to identify regional patterns and to highlight countries that were leading or lagging in digital maturity or primary health care performance. Further, to evaluate countries’ relative efficiency or inefficiency in translating health spending into a mature digital ecosystem or good primary health care, we mapped digital maturity and performance index values against current health expenditure per capita in 2019, measured in US$.^50^

We used two linear regression models to assess the relationship between the digital maturity index and current health expenditure per capita and between the performance index and current health expenditure per capita, respectively (details available in the data repository).^32^ Both models used log-transformed values for current health expenditure to reduce data skewness. From the first model, we derived predicted digital maturity index values for countries from their current health expenditure. From the second model, we derived predicted performance index values for countries from their current health expenditure. We validated both models using the Shapiro–Wilk test of normality.^51^ Predicted values for a country’s digital maturity and primary health care performance indices were calculated using the formula:
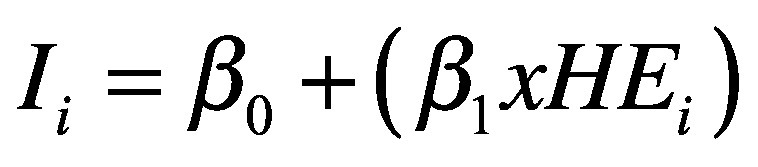
(1)where *I* is the predicted digital maturity or performance index; *i* is country of interest; *β_0_* is the intercept; *β_1_* represents the slope or rate of change in the predicted value for a one-unit increase in current health expenditure per capita; and *HE_country_* is the current health expenditure per capita by the country.

We then conducted a residual analysis to examine discrepancies between actual and predicted digital maturity and performance index values (details available in the online repository).^32^ In short, the residual digital maturity or performance index value was calculated as the observed value minus the corresponding predicted value. By plotting residual digital maturity index values against residual performance index values, we were able to divide countries into four quadrants based on high or low residual index values: (i) high residual digital maturity and performance index values (i.e. both >  0); (ii) high residual digital maturity and low residual performance index values (i.e. >  0 and <  0, respectively); (iii) low residual digital maturity and high residual performance index values (i.e. <  0 and >  0, respectively); and (iv) low residual digital maturity and performance index values (i.e. both <  0).

We performed all analyses using Excel (Microsoft Corporation, Redmond, United States of America); R v. 4.2.2 (The R Foundation, Vienna, Austria); and Stata v. 16.1 (StataCorp LLC, College Station, USA).

## Results

**Fig. 1:**
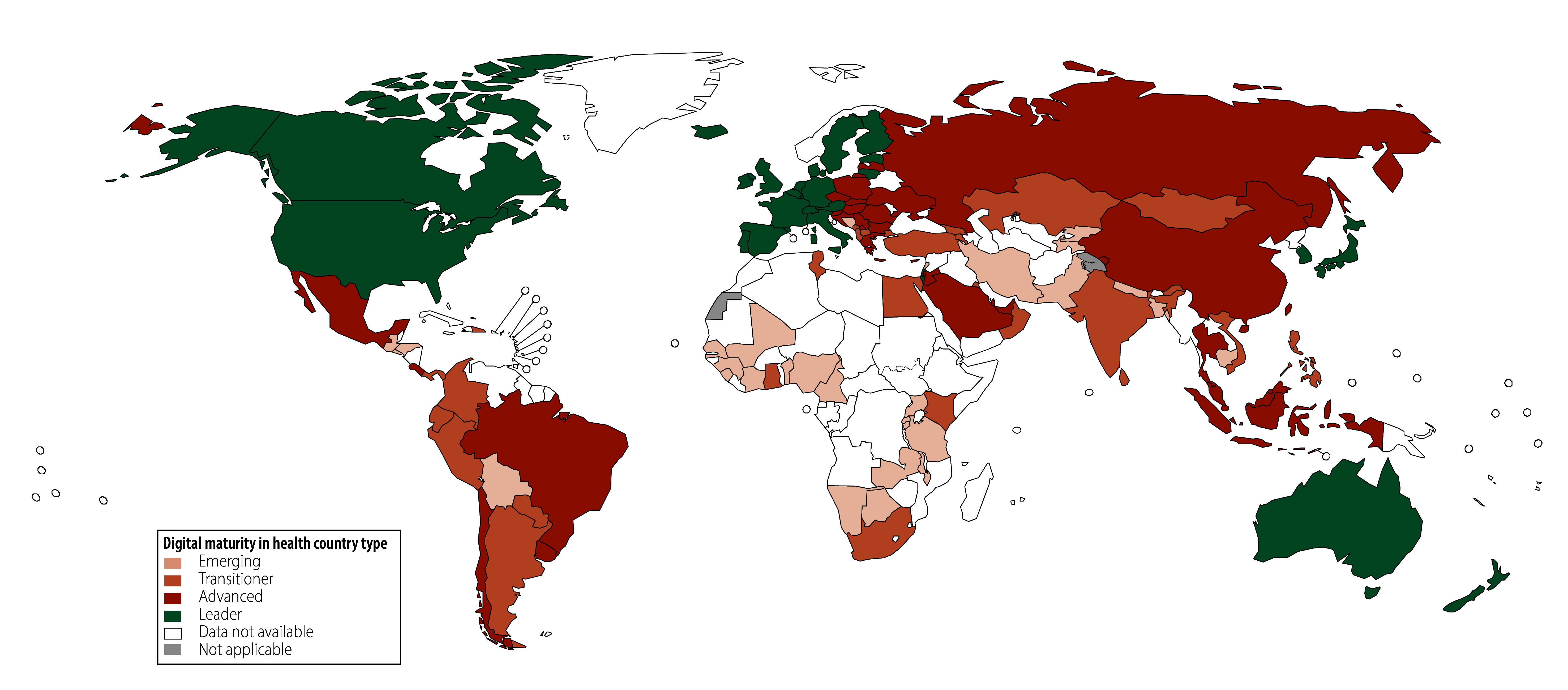
Country types of the digital maturity in health index, 109 countries, 2023

Notes: Countries with data available on digital maturity in health were divided into four types corresponding to quartiles of their digital maturity in health index: (i) emerging (index: 27.9 to 60.3); (ii) transitioner (index: 60.4 to 69.8); (iii) advanced (index: 69.9 to 78.3); and (iv) leader (index: 78.4 to 92.7). The category designated not applicable refers to disputed territories.

Of the 194 WHO Member States assessed, 85 (43.8%) had missing data on digital maturity (Fig. 1): the highest proportion was in the WHO African Region (59.6%, 28/47), followed by the Western Pacific Region (59.3%, 16/27), the Region of the Americas (48.6%, 17/35), the Eastern Mediterranean Region (47.6%, 10/21), the South-East Asia Region (45.5%, 5/11) and the European Region (17.0%, 9/53). The subcomponent with the highest proportion of missing data (39.7%; 77/194) was legislation, policy and compliance, whereas leadership and governance had the smallest proportion (1.0%; 2/194). The distribution of missing data is shown in the data repository.^32^

An analysis of the 109 countries with complete data available showed that the WHO region with the highest digital maturity was the European Region (average index: 76.5), followed by the Western Pacific Region (average index: 74.2), the Region of the Americas (average index: 67.6), the Eastern Mediterranean Region (average index: 65.3), the South-East Asia Region (average index: 61.5) and the African Region (average index: 51.2). Most African countries were categorized as emerging, except Ghana, Kenya, Mauritius and South Africa, which were categorized as transitioners. In the Region of the Americas, most countries, excluding Canada and the United States, were emerging or transitioners; with Brazil, Chile, Costa Rica, Mexico and Uruguay categorized as advanced. In the Eastern Mediterranean Region, Jordan, Qatar, Saudi Arabia and the United Arab Emirates were advanced among mostly emerging and transitioner countries. Indonesia and Thailand were categorized as advanced in the South-East Asia Region, among mostly emerging or transitioner countries. The European Region had mostly advanced or leader countries, with Bosnia and Herzegovina, Kyrgyzstan and Tajikistan categorized as emerging; and Albania, Armenia, Georgia, Kazakhstan, Montenegro, North Macedonia and Türkiye categorized as transitioners. Among the mostly advanced and leader countries in the Western Pacific Region, Cambodia was identified as emerging; and Mongolia, the Philippines and Viet Nam were categorized as transitioners.

### Regional patterns

**Fig. 2:**
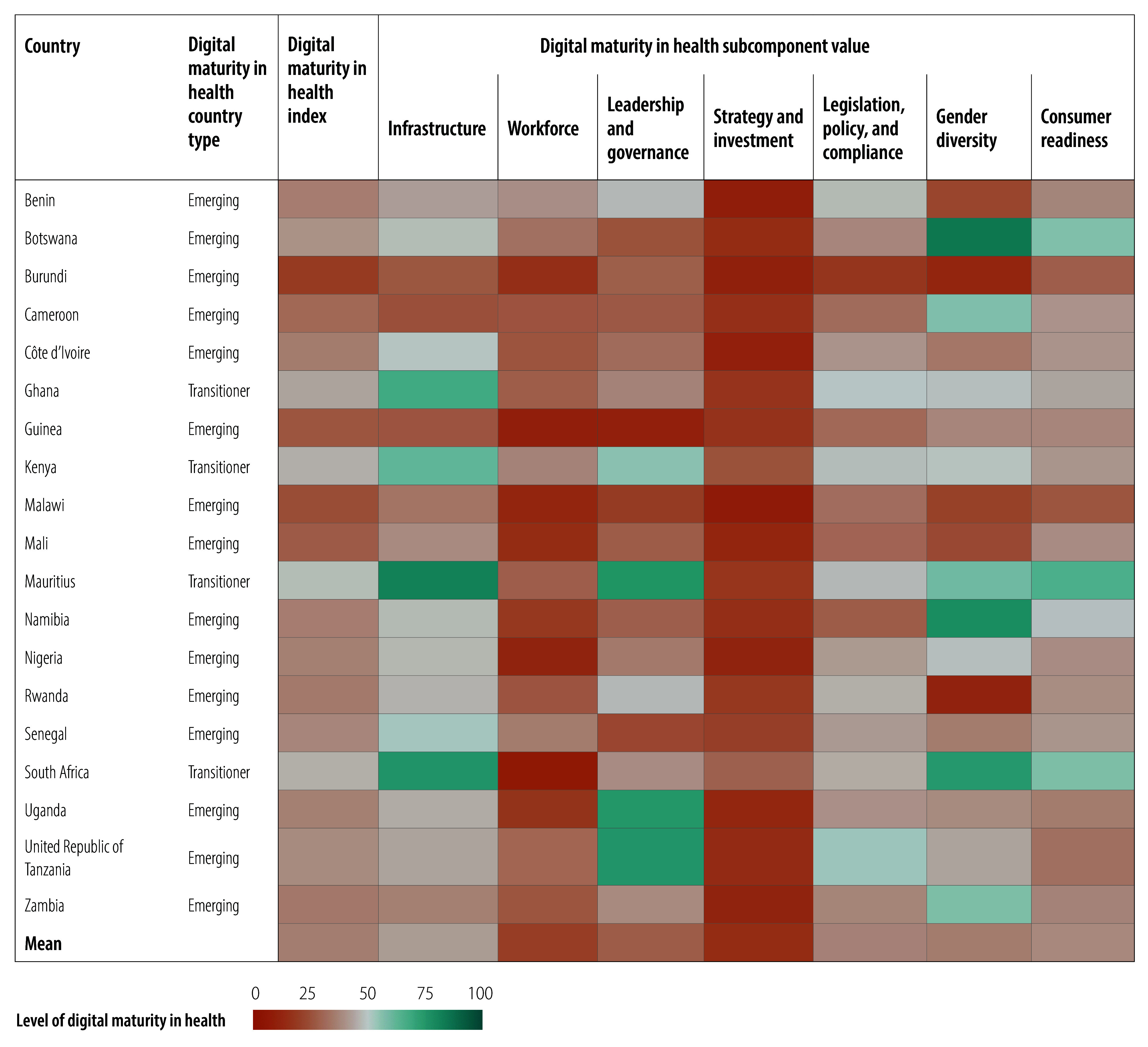
Subcomponent values of the digital maturity in health index by country with data in the WHO African Region, 2023

**Fig. 3:**
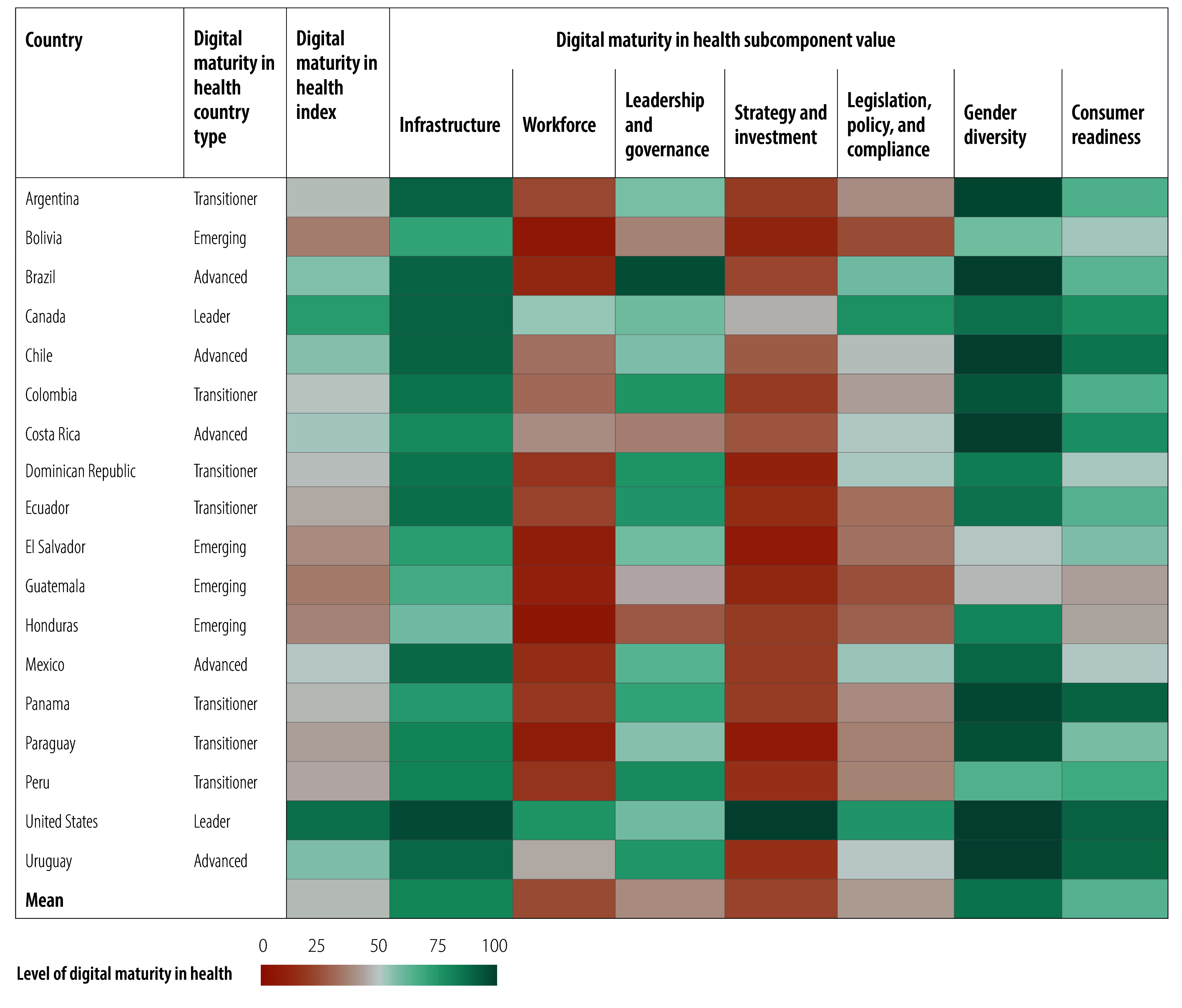
Subcomponent values of the digital maturity in health index by country with data in the WHO Region of the Americas, 2023

**Fig. 4:**
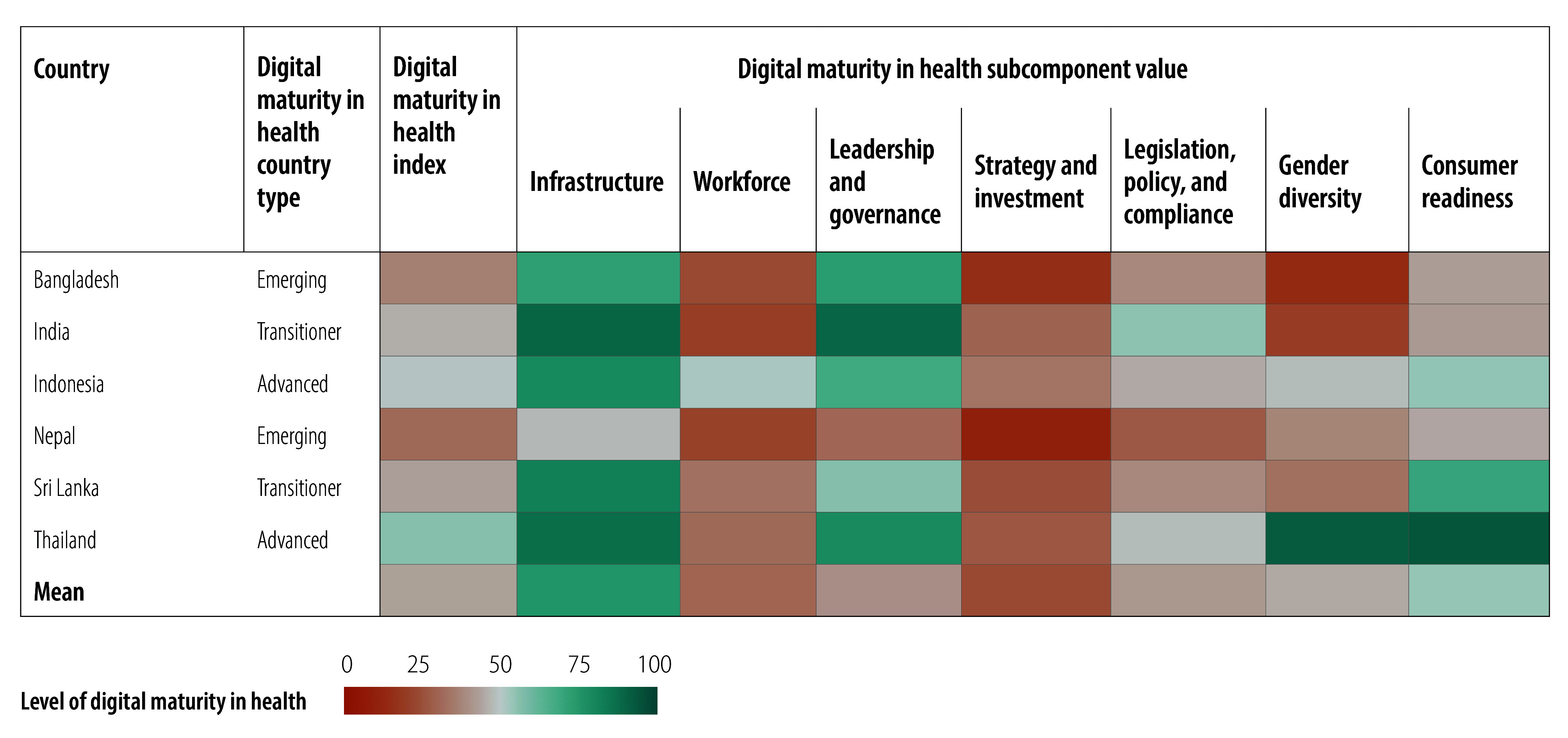
Subcomponent values of the digital maturity in health index by country with data in the WHO South-East Asia Region, 2023

**Fig. 5:**
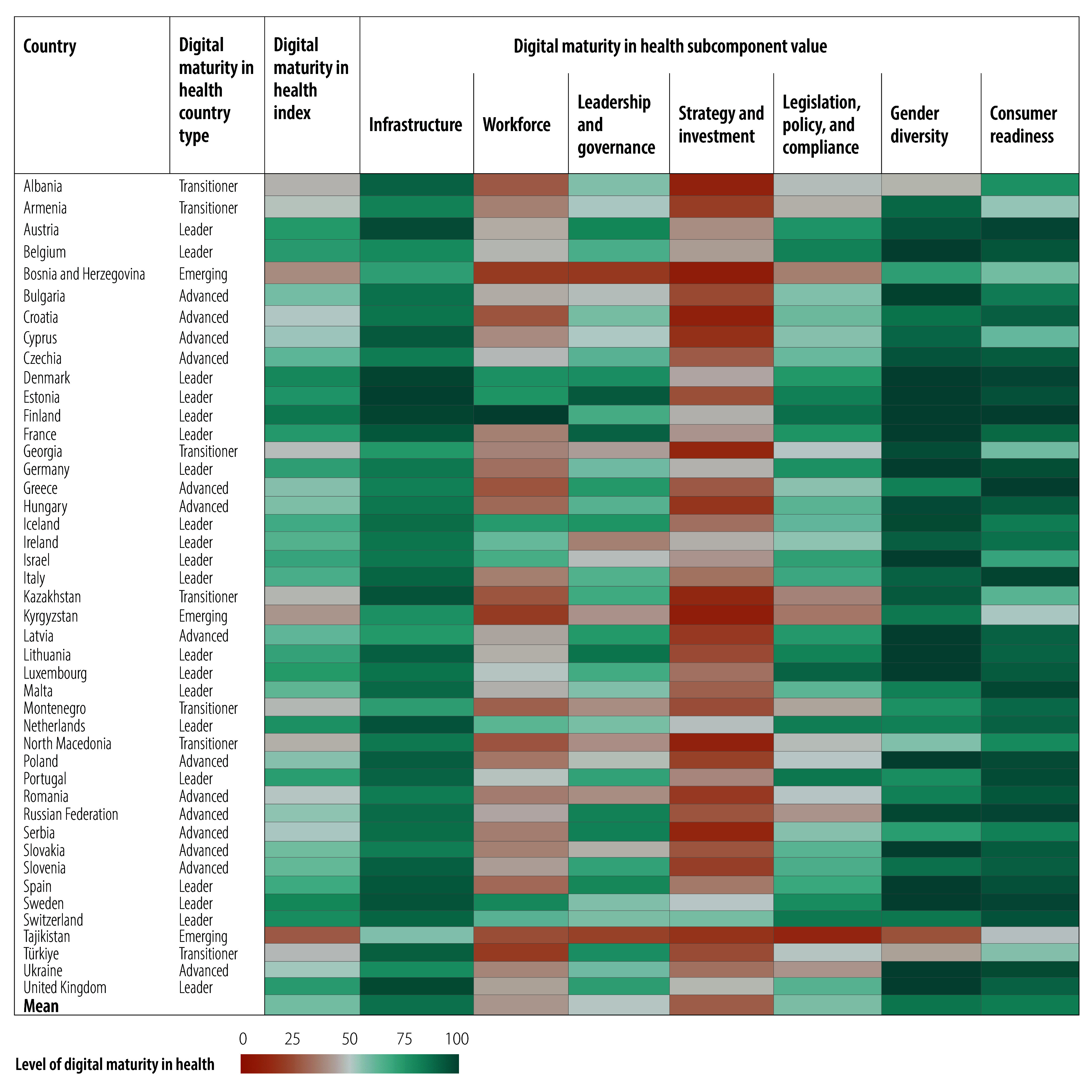
Subcomponent values of the digital maturity in health index by country with data in the WHO European Region, 2023

**Fig. 6:**
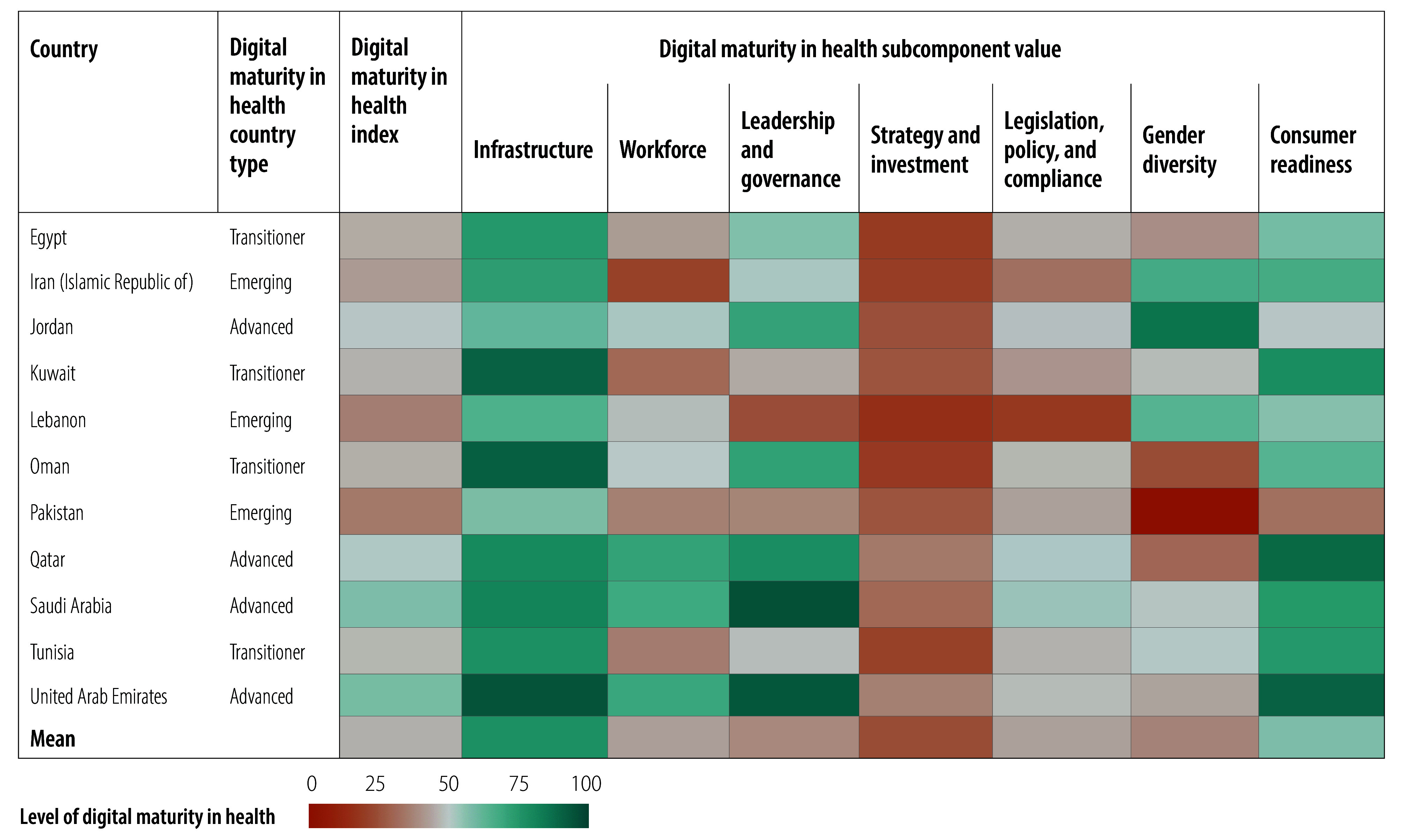
Subcomponent values of the digital maturity in health index by country with data in the WHO Eastern Mediterranean Region, 2023

**Fig. 7:**
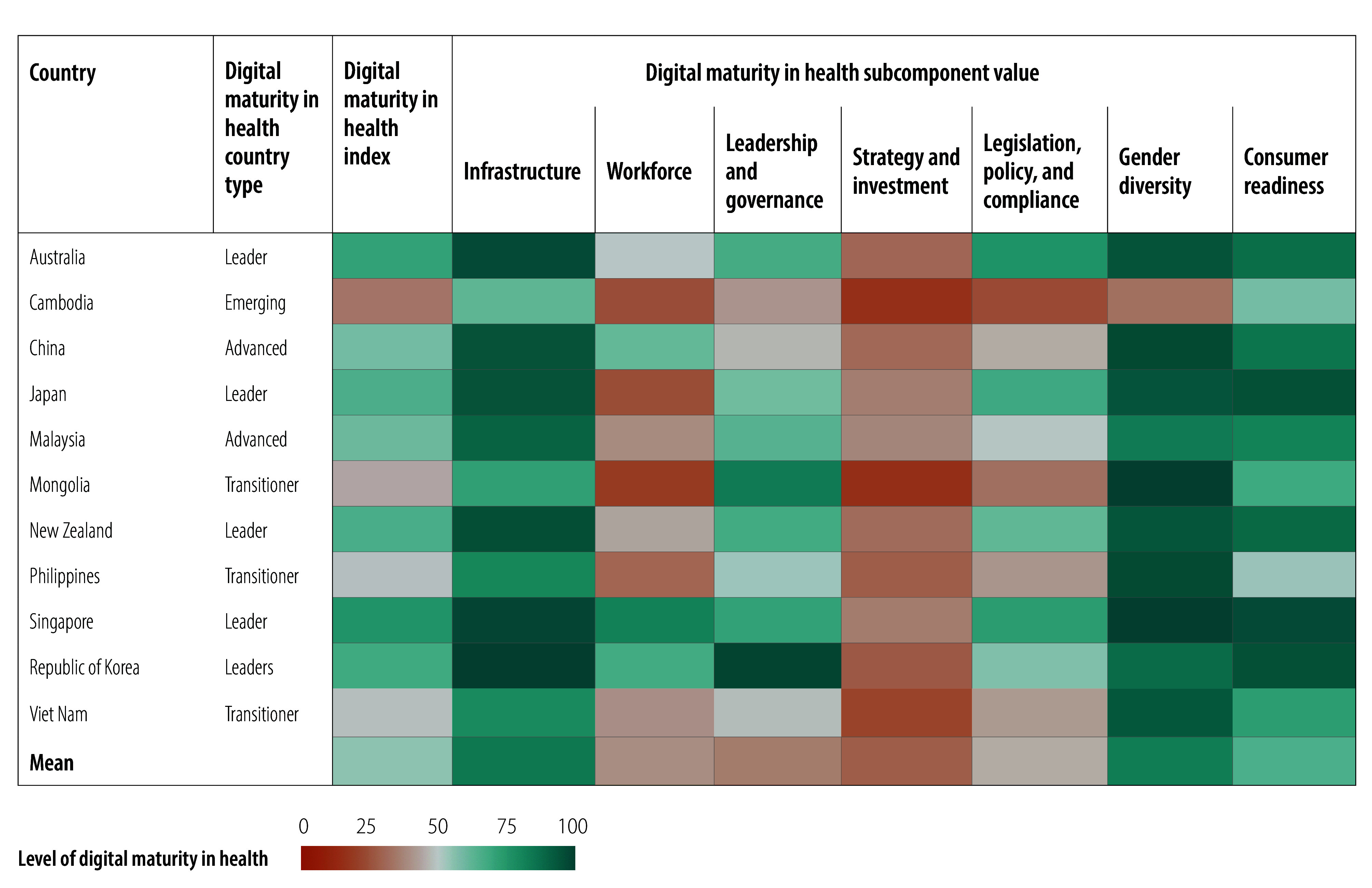
Subcomponent values of the digital maturity in health index by country with data in the WHO Western Pacific Region, 2023

Relative values for the digital maturity index and subcomponents of the 109 countries are illustrated in Fig. 2, Fig. 3, Fig. 4, Fig. 5, Fig. 6 and Fig. 7 (numerical values for each country are available in the online repository),^32^ from which leading and lagging countries in specific areas can be identified in each region. For example, the United Republic of Tanzania, categorized as an emerging country with an overall digital maturity index of 55.8, had a high leadership and governance subcomponent value of 86.0. In contrast, Japan (a leader country) and Brazil (an advanced country) had digital maturity index values of 80.2 and 75.1, respectively, but low workforce subcomponent values of 35.8 and 19.5, respectively. The ranked digital maturity index values for all 109 countries are reported in the online repository.^32^

WHO: World Health Organization.

Notes: Digital maturity in health subcomponent values were the averages of the individual indicators constituting each subcomponent; and the digital maturity in health index was the average composite score across all 14 indicators normalized on scales of 0–100 (Table 1). Countries were divided into four types corresponding to quartiles of their index: (i) emerging (index: 27.9 to 60.3); (ii) transitioner (index: 60.4 to 69.8); (iii) advanced (index: 69.9 to 78.3); and (iv) leader (index: 78.4 to 92.7).

WHO: World Health Organization.

Notes: Digital maturity in health subcomponent values were the averages of the individual indicators constituting each subcomponent; and the digital maturity in health index was the average composite score across all 14 indicators normalized on scales of 0–100 (Table 1). Countries were divided into four types corresponding to quartiles of their index: (i) emerging (index: 27.9 to 60.3); (ii) transitioner (index: 60.4 to 69.8); (iii) advanced (index: 69.9 to 78.3); and (iv) leader (index: 78.4 to 92.7).

WHO: World Health Organization.

Notes: Digital maturity in health subcomponent values were the averages of the individual indicators constituting each subcomponent; and the digital maturity in health index was the average composite score across all 14 indicators normalized on scales of 0–100 (Table 1). Countries were divided into four types corresponding to quartiles of their index: (i) emerging (index: 27.9 to 60.3); (ii) transitioner (index: 60.4 to 69.8); (iii) advanced (index: 69.9 to 78.3); and (iv) leader (index: 78.4 to 92.7).

WHO: World Health Organization.

Notes: Digital maturity in health subcomponent values were the averages of the individual indicators constituting each subcomponent; and the digital maturity in health index was the average composite score across all 14 indicators normalized on scales of 0–100 (Table 1). Countries were divided into four types corresponding to quartiles of their index: (i) emerging (index: 27.9 to 60.3); (ii) transitioner (index: 60.4 to 69.8); (iii) advanced (index: 69.9 to 78.3); and (iv) leader (index: 78.4 to 92.7).

WHO: World Health Organization.

Notes: Digital maturity in health subcomponent values were the averages of the individual indicators constituting each subcomponent; and the digital maturity in health index was the average composite score across all 14 indicators normalized on scales of 0–100 (Table 1). Countries were divided into four types corresponding to quartiles of their index: (i) emerging (index: 27.9 to 60.3); (ii) transitioner (index: 60.4 to 69.8); (iii) advanced (index: 69.9 to 78.3); and (iv) leader (index: 78.4 to 92.7).

WHO: World Health Organization.

Notes: Digital maturity in health subcomponent values were the averages of the individual indicators constituting each subcomponent; and the digital maturity in health index was the average composite score across all 14 indicators normalized on scales of 0–100 (Table 1). Countries were divided into four types corresponding to quartiles of their index: (i) emerging (index: 27.9 to 60.3); (ii) transitioner (index: 60.4 to 69.8); (iii) advanced (index: 69.9 to 78.3); and (iv) leader (index: 78.4 to 92.7).

### Subcomponent distributions

**Fig. 8:**
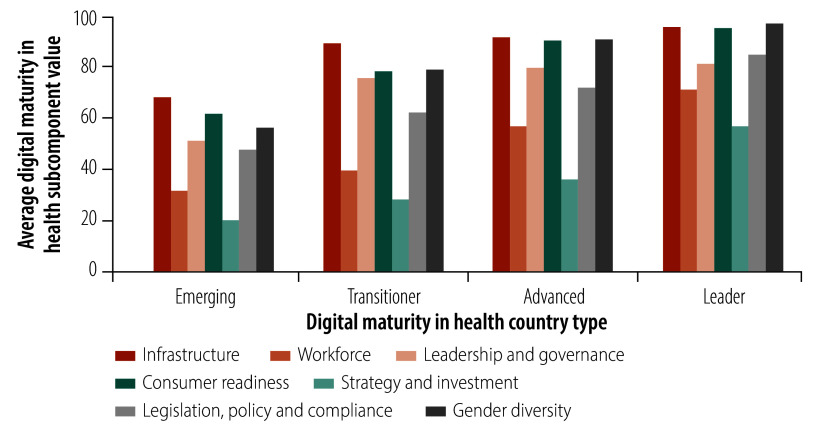
Digital maturity in health subcomponent values, by digital maturity in health country type, 109 countries, 2023

Notes: Digital maturity in health subcomponent values were the averages of the individual indicators constituting each subcomponent normalized on scales of 0–100 (Table 1). Countries were divided into four types corresponding to quartiles of their digital maturity in health index: (i) emerging (index: 27.9 to 60.3); (ii) transitioner (index: 60.4 to 69.8); (iii) advanced (index: 69.9 to 78.3); and (iv) leader (index: 78.4 to 92.7).

We found that leader countries had the highest values for all subcomponents compared to the other country types (Fig. 8). For leader countries, the gender diversity, infrastructure and consumer readiness subcomponents had the highest values. For emerging, transitioner and advanced countries, infrastructure was the strongest subcomponent. Across all country types, the subcomponent with the lowest value was strategy and investment. Subcomponents with high values across all country types were consumer readiness (that is, mobile phone ownership), leadership and governance, and gender diversity. The distributions of subcomponent values by WHO region and digital maturity country type are reported in the online repository.^32^

### Digital maturity versus UHC performance

**Fig. 9:**
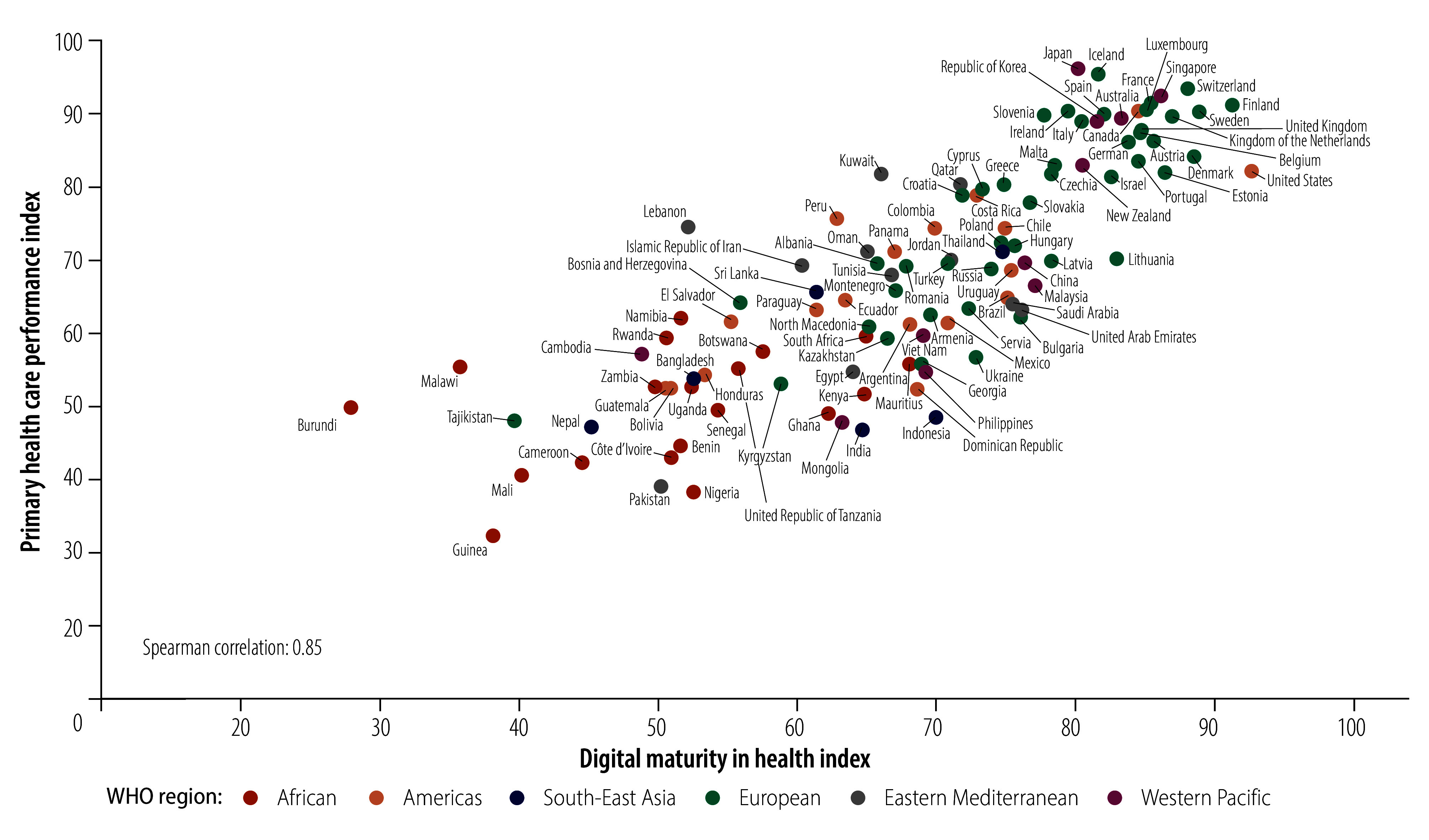
Primary health care performance index versus digital maturity in health index, 109 countries, 2023

There is a strong, positive, nonlinear correlation between the performance index and the digital maturity index (Spearman correlation *ρ* : 0.85; 95% confidence interval, CI: 0.77 to 0.90; Fig. 9). As the digital maturity index increased, the performance index rose slowly at first, then more rapidly, before levelling off. However, this correlation does not imply a direct causal relationship between digital maturity and primary health care performance. For example, Burundi, Lebanon and Malawi had low digital maturity index values but high performance index values; and India, Indonesia and Nigeria had high digital maturity index values but low performance index values. Further information on the correlation between digital maturity and performance index values, stratified by WHO region and digital maturity country type, are provided in the online repository.^32^

WHO: World Health Organization.

Notes: The primary health care performance index was the universal health coverage effective coverage index developed by the Institute for Health Metrics and Evaluation.^48^ It is a composite of 23 indicators across various health service areas normalized on scales of 0–100. The digital maturity in health index was the average composite score across 14 indicators normalized on scales of 0–100 (Table 1). The best-fit curve was based on a generalized additive model with a Gaussian distribution and an identity link function.

### Effect of health spending

We assessed the efficiency of health spending on improved digital maturity or primary health care performance by plotting digital maturity and performance index values, respectively, against log-transformed current health expenditure per capita (details available in the data repository).^32^ Countries above the resulting best-fit lines were regarded as having high digital maturity or primary health care performance for their current health expenditure per capita, whereas those below these lines had low digital maturity or primary health care performance for their current health expenditure per capita. For example, Romania had a digital maturity index of 70.8 compared with 52.1 for Lebanon despite similar current health expenditure per capita: US$ 738.6 and US$782.2, respectively. Moreover, a comparison of the two countries with highest health spending found that Switzerland had a performance index of 93.5 for US$ 9341.1 current health expenditure per capita, whereas the United States had a performance index of 82.1 for US$ 10 658.4 current health expenditure per capita.

**Fig. 10:**
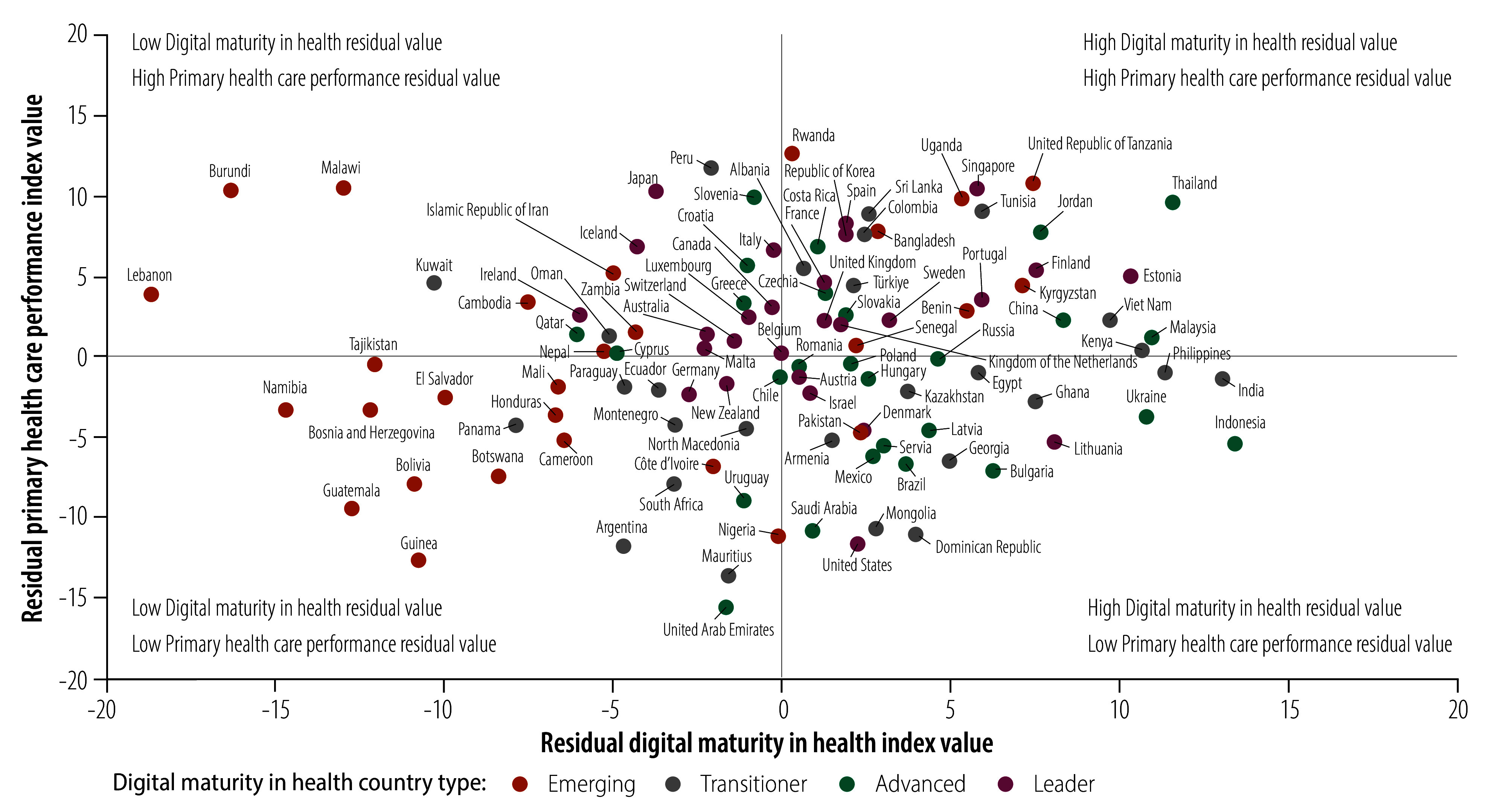
Primary health care performance versus digital maturity in health relative to current health expenditure, by digital maturity in health country type, 109 countries, 2023

Notes: The residual primary health care performance index value was calculated as the observed value minus the corresponding value predicted from the country’s current health expenditure. Similarly, the residual digital maturity in health index value was calculated as the observed value minus the corresponding value predicted from the country’s current health expenditure. Countries were divided into four types corresponding to quartiles of their digital maturity in health index: (i) emerging (index: 27.9 to 60.3); (ii) transitioner (index: 60.4 to 69.8); (iii) advanced (index: 69.9 to 78.3); and (iv) leader (index: 78.4 to 92.7). The figure is divided into four quadrants: (i) high residual digital maturity in health and primary health care performance index values (i.e. both > 0; top right quadrant); (ii) high residual digital maturity in health and low residual primary health care performance index values (i.e. > 0 and <  0, respectively; bottom right quadrant); (iii) low residual digital maturity in health and high residual primary health care performance index values (i.e. <  0 and >  0, respectively; top left quadrant); and (iv) low residual digital maturity in health and primary health care performance index values (i.e. both <  0; bottom left quadrant).

**Fig. 11:**
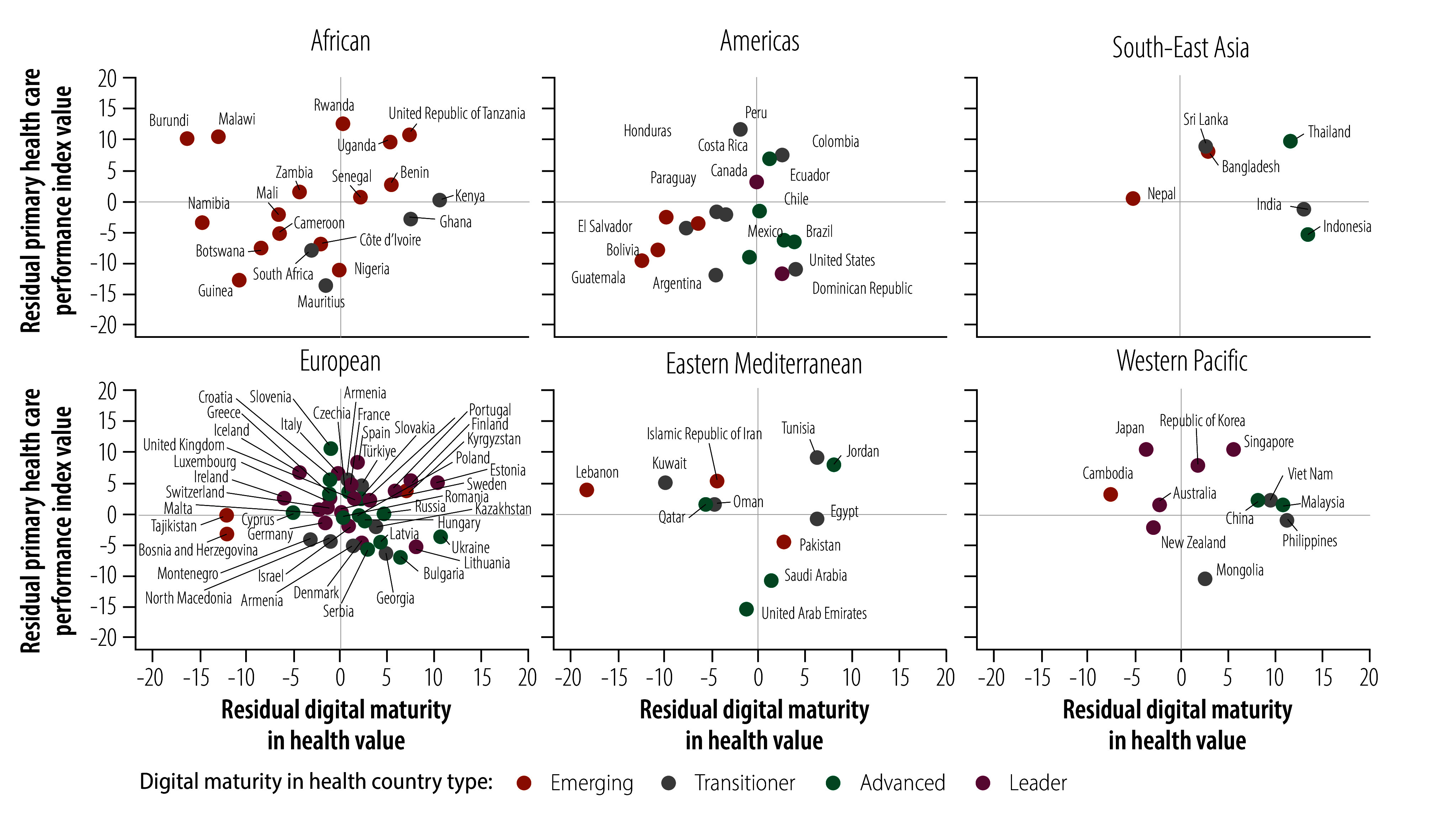
Primary health care performance versus digital maturity in health relative to current health expenditure, by WHO region and digital maturity in health country type, 109 countries, 2023

To examine associations between the efficiency of countries’ health spending on improved digital maturity and the efficiency of spending on improved primary health care performance, we plotted countries’ residual digital maturity index values against their residual performance index values, where the residual index value was the observed value minus the corresponding value predicted from the country’s current health expenditure (details available in the online repository).^32^ The resulting scatter plot was divided into four quadrants (Fig. 10). Most countries in the upper right quadrant, which indicates high health spending efficiency for both digital maturity and primary health care performance, are advanced or leader countries, but a few emerging and transitioner countries were included. The lower left quadrant, which indicates low health spending efficiency for both digital maturity and primary health care performance, contains mostly emerging and transitioner countries, with a few advanced and leader countries. Fig. 11 (available at https://www.who.int/publications/journals/bulletin/) displays the equivalent scatter plots for the six WHO regions. The corresponding scatter plots for different digital maturity country types are provided in the online repository.^32^

WHO: World Health Organization.

Notes: The residual primary health care performance index value was calculated as the observed value minus the corresponding value predicted from the country’s current health expenditure. Similarly, the residual digital maturity in health index value was calculated as the observed value minus the corresponding value predicted from the country’s current health expenditure. Countries were divided into four types corresponding to quartiles of their digital maturity in health index: (i) emerging (index: 27.9 to 60.3); (ii) transitioner (index: 60.4 to 69.8); (iii) advanced (index: 69.9 to 78.3); and (iv) leader (index: 78.4 to 92.7). Each figure is divided into four quadrants: (i) high residual digital maturity in health and primary health care performance index values (i.e. both > 0; top right quadrant); (ii) high residual digital maturity in health and low residual primary health care performance index values (i.e. > 0 and <  0, respectively; bottom right quadrant); (iii) low residual digital maturity in health and high residual primary health care performance index values (i.e. <  0 and >  0, respectively; top left quadrant); and (iv) low residual digital maturity in health and primary health care performance index values (i.e. both <  0; bottom left quadrant).

## Discussion

We found that high digital maturity generally aligned with high primary health care performance. However, some countries had high digital maturity without high primary health care performance and vice versa. This finding suggests that, although digital maturity can enhance primary health care functioning,^15^^,^^52^^,^^53^ it is not necessary for its improvement. In addition to providing insights into the relationship between digital maturity and primary health care performance, our study also identified problems with the availability and quality of data on digital maturity and highlighted the need for further research as countries develop their digital health strategies.

Data on digital maturity were missing for more than two fifths of WHO Member States, with the WHO African Region having the highest proportion. The missing data were not evenly distributed across subcomponents; legislation, policy and compliance had the highest rate of missing data, followed by strategy and investment. As countries develop their health sectors, timely and comprehensive data are crucial for monitoring progress and guiding investment. Our analysis provided data to supplement the digital health maturity profiles recorded by the Global Digital Health Monitor,^54^ which covered fewer countries at the time of our study. Moreover, for countries with data included by the Global Digital Health Monitor, our findings were in alignment with Global Digital Health Monitor indicators 2a, 4, 6a, 7, 8, 17 and 18.^55^

Our analysis of digital maturity country types demonstrated that some countries in the African Region had a high level of digital maturity, which other African countries with similar health systems could potentially learn from. The strongest subcomponents across all country types were infrastructure, gender diversity and consumer readiness, which correspond with global trends in mobile phone connectivity and access to electricity,^38^^,^^56^ along with the increasing ubiquity of mobile phone ownership, especially among women.^55^^,^^57^ These subcomponents are known to be crucial for the effective adoption of digital health programmes. For instance, researchers showed that connectivity and technical problems hindered the success of a mobile health programme in Ghana.^58^

We found that the relationship between health-care expenditure and digital maturity and primary health care performance varied widely among countries with similar spending and digital maturity country types. Although most countries in which health-care expenditure was associated with high digital maturity and primary health care performance were categorized as advanced or leader countries, expenditure was also used efficiently by some emerging and transitioner countries. Conversely, expenditure was not used efficiently by many emerging and transitioner countries and by a few advanced and leader countries. Valuable policy insights into improving the efficient use of health-care expenditure could be gained from countries with high digital maturity relative to spending (for example, India, Indonesia and Thailand) and from countries with high primary health care performance relative to spending (for example, Rwanda, Peru and the United Republic of Tanzania). Consequently, strategies for improving primary health care must be context-specific and consider health system structures, leadership and resource distribution as well as the level of digital maturity.

Our study had several limitations. First, the absence of data on digital maturity from many Member States limits the generalizability of our findings to countries with adequate resources and robust digital ecosystems. Second, the two subcomponents (i) standards and interoperability; and (ii) services and applications, which are regarded as important by the WHO and the International Telecommunication Union’s eHealth strategy toolkit,^31^ were excluded from our analysis due to a lack of measurable indicators. Moreover, several indicators used to measure digital maturity were geared towards the maturity of the digital ecosystem in general and did not have a focus on health. Although they are relevant to digital maturity, they remain suboptimal. Measures of digital maturity should be validated and expanded to encompass indicators related to (i) spending on digital applications and services in health care; (ii) the adoption of health-specific digital applications (e.g. electronic health records and telemedicine platforms) and services (e.g. electronic registries, health information exchanges and terminology services); (iii) the interoperability of, and standards for, digital systems; and (iv) the adoption and use of digital systems by both health workers and patients for health-related purposes. Finally, our study's ecological and cross-sectional design limited our ability to infer causality between digital maturity, primary health care performance and health-care expenditure.

In conclusion, our global study revealed countries with a high level of digital maturity, from which valuable insights and strategies could be learnt. In addition, by identifying countries lagging in digital maturity and weaker subcomponents, we highlighted areas requiring increased investment in digital health. Our findings are particularly relevant for the Global Initiative in Digital Health, which aims to identify and allocate resources to underfunded areas of digital health.^59^ Moreover, our study shows that the systematic identification and monitoring of core components of digital health ecosystems is essential for driving the digital transformation of health systems and enhancing the accessibility, quality and equity of health care worldwide.
